# Measles Vaccination and Outbreaks in Croatia from 2001 to 2019; A Comparative Study to Other European Countries

**DOI:** 10.3390/ijerph19074140

**Published:** 2022-03-31

**Authors:** Ines Drenjančević, Senka Samardžić, Ana Stupin, Katalin Borocz, Peter Nemeth, Timea Berki

**Affiliations:** 1 Institute and Department of Physiology and Immunology, Faculty of Medicine Osijek, Josip Juraj Strossmayer University of Osijek, 31000 Osijek, Croatia; ana.stupin@mefos.hr; 2 Croatian National Scientific Center of Excellence for Personalized Health Care, Josip Juraj Strossmayer University of Osijek, 31000 Osijek, Croatia; 3 Teaching Institute of Public Health of the Osijek-Baranya County, 31000 Osijek, Croatia; 4 Clinical Centre, Department of Immunology and Biotechnology, University of Pécs Medical School, 7624 Pécs, Hungary; borocz.katalin@pte.hu (K.B.); nemeth.peter@pte.hu (P.N.); berki.timea@pte.hu (T.B.)

**Keywords:** measles-rubella-mumps vaccination, Europe, Croatia

## Abstract

Due to the current burden of COVID-19 on public health institutions, increased migration and seasonal touristic traveling, there is an increased risk of epidemic outbreaks of measles, mumps and rubella (MMR). The aim of the present study was to analyze the epidemiological data on MMR immunization coverage and the number of measles cases in 2001–2019 in Croatia and a number of European countries. Results revealed a decreasing trend in vaccination in 2001–2019 throughout Europe. However, Croatia and Hungary still have the highest primary and revaccination coverage, compared to other analyzed countries. The highest number of measles cases was in 2017 in Romania. There was no significant correlation between the percentage of primary vaccination and the number of measles cases (r = −0.0528, *p* = 0.672), but there was a significant negative correlation between the percentage of revaccination and the number of measles cases (r = −0.445, *p* < 0.0001). In conclusion, the results of the present study emphasize the necessity to perform a full protocol of vaccination to reach appropriate protection from potential epidemic outbreaks. Furthermore, in the light of present migrations, documenting the migrants’ flow and facilitating vaccination as needed is of utmost importance to prevent future epidemics.

## 1. Introduction

Throughout the world, vaccination with safe, effective, and affordable vaccines for measles, mumps and rubella (MMR) is freely available. The measles vaccination was introduced into the compulsory vaccination program in the Republic of Croatia in 1969. The first vaccination effort vaccinated all children one to six years old. Vaccination against rubella (1975) and mumps (1976) soon followed [1]. MMR vaccination began in 1976. Since the beginning of vaccination against MMR, a vaccine of domestic production (Immunology Institute) has been used. The measles vaccine strain, Edmonston–Zagreb, as well as the rubella vaccine strain, RA 27/3, were produced on human diploid cell culture, while the mumps virus vaccine strain was produced on chicken fibroblast cell culture [2,3]. The use of vaccines against these diseases has led to their almost complete eradication in Croatia, with sporadic cases. The Croatian law prescribes the required minimum coverage for measles vaccination of 95%. In Croatia, outbreaks of measles occurred in 2015 and 2018. In 2018, an outbreak in the southern-Adriatic part of the country was a consequence of the infection of an adult returning from Kosovo, with 15 epidemiologically-linked cases [4]. The median age of infected persons was 33 years, while one case was an 8-month-old infant. Two of these cases had received two doses of a measles-containing vaccine, one person had taken one dose and three were unvaccinated, while for nine cases, vaccination status was unknown [4]. In regard to neighboring countries, in 2017, there was a small outbreak of measles in Hungary, in close proximity of Osijek-Baranja County, which was not spread wider over the state border due to good epidemiological measures [5]. Measles outbreaks in 2018–2019 in the Croatian cities of Zagreb, Slavonski Brod, Split and Dubrovnik demonstrated possibly suboptimal vaccination coverage in certain cluster(s) of the population [5]. Despite the proximity of Slavonski Brod (tens of kilometers) and frequent commutation between counties, Osijek-Baranja County was not affected.

In the light of flaming waves of the COVID-19 pandemic, other contagious diseases somehow were put aside, partly due to successful vaccination programs, particularly in European countries. In the period between the end of 2019 and the spring of 2022, the COVID-19 pandemic significantly influenced interpersonal contacts, which also have different impacts on measles, mumps and rubella vaccination efforts and burden across the world. For example, Brazil reported a reduction in the number of MMR vaccine doses [6], while interestingly, Japan reported de-creased estimated annual burdens in 2020 for measles (98%), mumps (47%) and rubella (94%) compared with those in 2019 due to social distances in COVID-19 pandemic [7]. However, due to the current burden of COVID-19 on public health institutions, one may expect a decrease in vaccination coverage and potential new outbreaks in near future. We hypothesized that there is a relationship between vaccination coverage and the number of measles cases. The present study aimed to analyze the epidemiological data on population immunization and reflect the number of measles cases in the 2001–2019 period for Croatia and countries of the European region.

## 2. Materials and Methods

Data collection was performed using European Centre for Disease Prevention and Control (ECDC) reports [8,9,10] and World Health Organization (WHO) statistics from 2001 to 2019 [11]. For Croatia, data from National Institute of Public Health and Ministry of Health was used (Croatian Health Service Yearbook for years 2001 to 2019 [12,13,14,15,16,17,18,19,20,21,22,23,24,25,26,27,28,29,30]).

Statistical analysis: Differences in percentage of vaccination (% vaccination) in observed period among regions/countries were analyzed using Two-way ANOVA, with appropriate post hoc test for multiple comparisons (Sidak’s or Tukey’s multiple comparisons test). Correlation between number of cases per year and vaccination coverage was assessed using Spearman’s correlation. *p* < 0.05 was considered statistically significant. GraphPad v6.0 (GraphPad Software, San Diego, CA, USA) and SigmaPlot, version 11.2 (Systat Software, Inc., Chicago, IL, USA) were used for statistical analysis.

## 3. Results

### 3.1. MMR Primary Vaccination and Revaccination in Croatia and Osijek-Baranja County 2001–2019

Table 1 presents data on the primary MMR vaccination in Croatia and particularly, Osijek-Baranja County (OBC). There was no significant difference in the percentage of primary vaccination between Croatia and OBC each year from 2001 to 2018. However, there was a significantly higher percentage of primary vaccination in Croatia compared to OBC only in 2019 (*p* < 0.05).

Table 2 presents the data on revaccination in Croatia and OBC. In Croatia, the percentage of revaccination compared to primary vaccination significantly increased in 2016 and 2017, with no significant change in other years between 2001 and 2019 (2016, *p* < 0.05; in 2017, *p* < 0.05). In OBC, the percentage of revaccination compared to primary vaccination significantly increased in 2009, 2010, 2016, 2017 and 2019 (*p* < 0.05), with no significant change in other years between 2001 and 2019.

### 3.2. MMR Primary Vaccination and Revaccination in Croatia and Neighboring Countries in Years 2001–2019

Table 3 presents differences in MMR primary vaccination among Croatia and neighboring countries. There was no significant difference in the percentage of primary vaccination between Croatia and neighboring countries from 2001 until 2015. Afterward, Croatia, Slovenia and Serbia generally had the best primary vaccination success, while Bosnia and Herzegovina (BiH) and North Macedonia had the lowest; e.g., in 2016, the percentage of primary vaccination in BiH was significantly lower compared to Croatia (2016, *p* < 0.05) and Slovenia (2016, *p* < 0.05), while in 2017 and 2018, the percentage of primary vaccination in BiH was significantly lower compared to Croatia (*p* < 0.05), Slovenia (*p* < 0.05), and Serbia (*p* < 0.05). Additionally, in 2018, North Macedonia had significantly lower percentage (75%) of primary vaccination compared to Croatia (2018, *p* < 0.05), Serbia (2018, *p* < 0.05), and Slovenia (2018, *p* < 0.05). Montenegro and the years 2013 and 2019 had to be excluded from the analysis due to missing data.

Table 4 presents the data on MMR revaccination among Croatia and neighboring countries. In 2001 and 2002, there was a significantly lower percentage of revaccination in Serbia compared to Croatia (*p* < 0.05). There was no significant difference in the percentage of revaccination among Croatia and neighboring countries in 2003–2005 and 2007–2017. In 2006, the percentage of revaccination in BiH was significantly lower compared to Croatia (*p* < 0.05), North Macedonia (in 2006, *p* < 0.05) and Serbia (in 2006, *p* < 0.05). In 2018, the percentage of revaccination in BiH was significantly lower compared to Croatia (2018 *p* < 0.05) North Macedonia (2018, *p* < 0.05) and Serbia (2018, *p* < 0.05). Montenegro and Slovenia and the years 2009, 2013 and 2019 had to be excluded from analysis due to missing data.

### 3.3. MMR Primary Vaccination and Revaccination in European Countries 2001–2019

Data for Table 5 and Table 6 cover Austria, Hungary, Croatia, Czech Republic, Denmark, Germany, Italy, Poland, France, Belgium and Ukraine.

Table 5 presents data on MMR primary vaccination in European countries. Austria and the Czech Republic and the years 2008, 2013, 2018 and 2019 had to be excluded for analysis because of partly missing data. In 2001, the percentage of primary vaccination in Italy was significantly lower compared to Hungary (*p* < 0.05), Croatia (*p* < 0.05), Denmark (*p* < 0.05) and Poland (*p* < 0.05). Furthermore, in 2001, Belgium had a significantly lower percentage of primary vaccination compared to Hungary (*p* < 0.05). In 2002, the percentage of primary vaccination in Italy was significantly lower compared to Hungary (*p* < 0.05), Denmark (*p* < 0.05) and Poland (*p* < 0.05). Moreover, in 2002, Belgium had a significantly lower percentage of primary vaccination compared to Hungary (*p* < 0.05), Denmark, (*p* < 0.05) and Poland (*p* < 0.05). In 2003 and 2004, Belgium had a significantly lower percentage of primary vaccination compared to Hungary (*p* < 0.05). In 2009, the percentage of primary vaccination in France was significantly lower compared to Hungary (*p* < 0.05), Croatia (*p* < 0.05), Germany (*p* < 0.05), Italy (*p* < 0.05), Poland (*p* < 0.05) and Belgium (*p* < 0.05). There was no significant difference in the percentage of primary vaccination between EU countries from 2005 until 2007 and from 2010 until 2017. According to available data, differences in the percentage of primary vaccination between Ukraine and other European countries were analyzed for the period between 2013 and 2017. In 2014, 2015 and 2016, Ukraine had a significantly lower percentage of primary vaccination compared to Hungary (*p* < 0.05), Croatia (*p* < 0.05), Denmark (*p* < 0.05), Germany (*p* < 0.05), Italy (*p* < 0.05), Poland (*p* < 0.05), France (*p* < 0.05) and Belgium (*p* < 0.05). There was no significant difference in the percentage of primary vaccination between Ukraine and Italy in 2013, and 2017, Austria and the Czech Republic were excluded from the analysis because of partly missing data.

Table 6 presents data on MMR revaccination in EU countries. Austria, Italy, France and Belgium and the years 2001, 2008, 2009 and 2019 had to be excluded from analysis due to missing data. In 2002, the percentage of revaccination in Germany was significantly lower compared to Hungary (2002, *p* < 0.05), Croatia (2002, *p* < 0.05), Czech Republic (2002, *p* < 0.05), Denmark (2002, *p* < 0.05) and Poland (2002, *p* < 0.05). In 2003 and 2004, the percentage of revaccination in Germany was significantly lower compared to Hungary (2003, *p* < 0.05; 2004, *p* < 0.05), Croatia (2003, *p* < 0.05; 2004, *p* < 0.05), the Czech Republic (2003, *p* < 0.05; 2004, *p* < 0.05) and Poland (2003, *p* < 0.05; 2004, *p* < 0.05.) There was no significant difference in percentage of revaccination between EU countries from 2005 until 2007 and from 2010 until 2018. Again, according to available data, differences in the percentage of revaccination between Ukraine and other European countries were analyzed for the period between 2013 and 2017. In 2013, 2014, 2015 and 2016, Ukraine had a significantly lower percentage of revaccination compared to Hungary—a particularly low percentage revaccination in 2016, only 31% (*p* < 0.05)—Croatia (*p* < 0.05), Czech Republic (*p* < 0.05), Denmark (*p* < 0.05), Germany (*p* < 0.05), Italy (*p* < 0.05), Poland (*p* < 0.05), France (*p* < 0.05) and Belgium (*p* < 0.05). There was no significant difference in the percentage of revaccination between Ukraine and other EU countries in 2017. Austria was excluded from analysis because of partly missing data.

Table 7 presents the number of measles cases in European countries, from 2001 to 2019. In 2006, 2018 and 2019, Ukraine had a significantly higher number of measles cases compared to all other European countries listed in Table 7. There was no significant difference in the number of measles cases among European countries in the periods between 2002 and 2005, 2007 and 2009, 2011 and 2017.

Analysis of the association between the percentage of primary vaccination and number of measles cases, just as between the percentage of revaccination and the number of measles cases between 2001 and 2019 included available data from the following countries: Austria, Hungary, Croatia, Czech Republic, Denmark, Germany, Italy, Poland, France, Belgium, Ukraine, Bosnia and Herzegovina, North Macedonia, Montenegro and Serbia. There was no significant correlation between the percentage of primary vaccination and the number of measles cases (r = −0.0671 *p* = 0.298) but there was a significant moderate negative correlation between the percentage of revaccination and the number of measles cases (r = −0.357 *p* < 0.0001).

## 4. Discussion

After the introduction of the measles vaccination, the number of affected patients decreased significantly. Before 1968 (when compulsory vaccination against measles was introduced in Croatia), the average annual number of patients in Croatia was around 15,000, while in the last ten years, this number has stayed below 20, with the exception of 2015, when we had an epidemic with 206 patients, and in 2018, with the measles epidemic in Dubrovnik-Neretva County [4]. Interestingly, our results show that in Croatia, the percentage of revaccination compared to primary vaccination significantly increased in 2016 and 2017. Analysis of neighboring countries of Croatia revealed that Croatia, Slovenia, and Serbia generally had the best primary vaccination success, while Bosnia and Herzegovina (BiH) and North Macedonia had the lowest primary vaccination percentage and BiH also had the lowest percentage of revaccination compared to neighboring countries (Table 3 and Table 4). Interestingly, in the first decade of the 21st century, Italy, Belgium and France had the lowest MMR primo-vaccination coverage of analyzed European countries. There was no significant difference in the percentage of primary vaccination between EU countries from 2005 until 2007 and from 2010 until 2017. However, in the period 2013–2017, Ukraine had the lowest primary vaccination and revaccination coverage compared to other European countries (e.g., in 2016 Ukraine had 47% primary vaccination and 31% revaccination). The highest percentages of coverage are seen in Hungary and Croatia (Table 5 and Table 6). This is in agreement with the notification rate per million population for measles. In Croatia from November 2020–October 2021 [8] and February 2021–January 2022, there were zero cases, and in Slovenia, Hungary, Slovakia, Czech Republic, Bulgaria, Greece, and Portugal, there were no cases of measles. Other EU countries reported 0.001–0.099 cases [9]. In contrast, in the period February 2020–January 2021, only Croatia, Hungary and Slovakia reported a notification rate per million of zero, while the majority of other EU countries had from 0.001–0.999 [10]. This could be attributed to suboptimal vaccine coverage in Europe, which led to a major resurgence of measles in recent years [32]. Several reasons may underline that situation, including increasing trends of vaccine hesitancy or refusal due to perception of measles risk and burden, mistrust in experts, concerns about vaccine safety, effectiveness, and accessibility [32]. Furthermore, migrations and consequences of wars or economical migrations from the countries with disturbed health care systems also influence vaccination coverage of the population. Importantly, one may hypothesize that the decrease in vaccination in the EU and neighboring countries increases the risk of an epidemic surge in the near future.

The biggest problem is the continuous decline in vaccination coverage of preschool children, which is below the minimum 95% and can lead to an epidemic [33]. Recently, in the study conducted in the frame of the CABCOS3 project, it was reported that the Hungarian serum samples and Croatian serum samples were largely overlapping in seropositivity ratios, which might be attributed to the intrinsic biological dynamics of vaccination-based humoral immunity to measles. Individuals 34–43 years old had the lowest seropositivity ratios (78%) [34]. A prospective study conducted in Prague, Czechia, on a total of 2782 participants aged 19–89 years, analyzed the level of measles-specific antibodies in serum samples and showed that the seropositivity rate in naturally immunized participants (before 54 years) was significantly higher than in fully vaccinated persons aged 19–48 (98.0% (95% CI: 96.5–99.0%) vs. 93.7% (95% CI: 92.4–94.9%)). Lower seropositivity persistence (86.6%) was found in a cohort of those born in 1971–1975, vaccinated mostly with one dose, compared to naturally immunized persons or compared to participants fully vaccinated with two doses [35]. Furthermore, in 2019, 59 measles cases were reported between 1 January and 11 March in Austria; 47 of them fulfilled the cluster case definition. Forty out of 47 patients (85.1%) were unvaccinated, while the age distribution of cases suggested measles immunity gaps in adults [36]. In Zagreb, Croatia, in the period from December 2014 to April 2015, 122 measles cases were notified, 93% of which were unvaccinated persons, age younger or equal to four years, and older than 20 [37]. The outbreak was successfully resolved, and Croatia has an excellent measles elimination profile [38]. Interestingly, in Korea, 2019, there were 26 measles case-patients, aged 18–28 years. Twenty-five of them had previously received the MMR vaccine (12/26, 46% (two doses); 13/26, 50% (one dose)), and 16 (62%) had positive results of measles IgG prior to measles diagnosis [39]. Altogether, these are important information in the light of the previously mentioned outbreak among adults in Dubrovnik-Neretva County [4], suggesting that the lack of previous immunization, together with a decrease in seropositivity, present a risk for future epidemic outbreaks.

It has been shown that several factors may influence the parental decision to choose MMR vaccination, such as confidence in experts and vaccine, measles severity, responsibility toward child and community health and peer judgment [32]. Through educational activities foreseen within CABCOS, our goal is to increase public awareness of the importance of vaccination and increase the share of vaccinated children. Trends of a decrease in immunization coverage are followed in other countries in the region. For example, in the years 2018 to 2020, in Kosovo, >90% (N = 430) of children 12–24 months old had fully completed immunization personal plans. There were delays in immunizations, from 1 to 3 months, mainly due to the COVID-19 pandemic, lack of time for parents to take the child for vaccination or the child being sick at the scheduled time of vaccination. The difference between non-vaccination and full vaccination was only related to the age of children (*p* < 0.001) [40].

In contrast to the situation in Croatia (Table 3 and Table 4), in Serbia, over the period 2000–2017, there was a significant decline in coverage of primary vaccination against measles, mumps, rubella (MMR) (*p* ≤ 0.01). In the same period, coverage of all subsequent revaccinations significantly decreased, e.g., in the second dose against MMR before enrolment in elementary school (*p* < 0.05) [41]. In Western Europe, the situation with vaccination coverage varies. 2018–2019 data in the UK, London area, showed that the coverage of children with dose two of MMR vaccine at their fifth birthday has been consistently low (76.3%) [42]. Results of the present study showed that Germany (Table 5 and Table 6) had also significantly lower primary vaccination and revaccination percentages compared to other countries in 2003 and 2004. Importantly, there was no significant difference in the percentage of revaccination between EU countries from 2005 until 2007 and from 2010 until 2018. It is not clear what was the cause of differences in immunization coverage in the period 2007 until 2010.

A recent systematic review (PROSPERO CRD42019157473; 1 January 2000 to 22 May 2020) identified studies on vaccine-preventable disease outbreaks involving migrants residing in the EU/EEA and Switzerland (including measles, mumps and rubella). 47 different vaccine-preventable disease outbreaks in 13 countries were reported in 45 studies. 40% of outbreaks (mostly varicella and measles) occurred in shelters or temporary refugee camps. Measles were the most reported outbreaks involving migrants (n = 24; 6496 cases) and 11 of them were associated with migrants from eastern European countries. There were only three reported rubella outbreaks (487 cases) and two reported mumps outbreaks (293 cases) [43]. As a study in 2017 demonstrated, the most important factor that prevented the resurgence of measles was vaccine coverage rates, regardless of the economic status of the country or the number of incoming travelers or migrants. In 2017, the incidence of measles was the highest in Romania (46.1/100,000), which has the lowest coverage rate (75%), followed by Ukraine (10.8/100,000) and Greece (8.7/100,000). Overall vaccination coverage with two doses in these countries was less than 84% [44]. Data from a 2017 survey on national immunization strategies to provide vaccinations for migrants show that Portugal, Italy, Croatia and Slovenia offer migrant children and adolescents all vaccinations included in the National Immunization Plan, and Greece and Malta provide only certain vaccinations, including those against measles–mumps–rubella and diphtheria–tetanus–pertussis and poliomyelitis. Portugal, Malta, Italy and Croatia also offer vaccination to adults. Vaccinations are delivered in holding centers and/or community health services in all countries. No country delivers vaccinations at the entry site to the country [45]. Thus, the finding of the present study, that there is a significant moderate negative correlation between the percentage of revaccination and the number of measles cases, provides additional support for the importance of the completion of vaccination protocols, since this correlation was not found in primo-vaccination.

## 5. Conclusions

In conclusion, the present study demonstrates that there is a negative correlation between the second vaccination (revaccination) and the number of measles cases, which emphasizes the necessity to perform a full protocol of vaccination to reach appropriate protection from potential epidemic outbreaks. Thus, it is important to have a strategy to document migrants’ flow and facilitate vaccination as needed; this is of utmost importance to prevent future epidemics. Additionally, follow-ups on seropositivity upon vaccination in the adult population should be monitored to highlight potential regions or sub-population at greater risk to be points of epidemic outbreaks.

## Figures and Tables

**Table 1:** Measles-containing vaccine primary vaccination in Osijek-Baranja County and Croatia from 2001 to 2019.

	Osijek-Baranja County Primary Vaccination			Croatia Primary Vaccination		
Year	Scheduled	Vaccinated	%	Scheduled	Vaccinated	%
2001	3312	3137	94.7	44,989	42,091	93.6
2002	3217	3001	93.3	44,467	42,110	94.7
2003	3019	2858	94.7	41,388	39,103	94.5
2004	2914	2755	94.5	40,985	39,230	95.7
2005	2849	2673	93.8	39,783	37,710	94.8
2006	3128	2984	95.4	41,721	39,797	95.4
2007	3032	2890	95.3	41,437	39,807	96.1
2008	2964	2826	95.3	41,714	39,855	95.5
2009	3030	2758	91.0	42,599	40,484	95.0
2010	3188	2934	92.0	44,659	42,855	96.0
2011	2992	2812	94.0	43,830	42,092	96.0
2012	2902	2682	92.4	41,606	39,446	94.8
2013	2928	2730	93.2	40,885	38,369	93.8
2014	2783	2605	93.6	39,862	37,342	93.7
2015	2300	2092	91.0	38,882	36,088	92.8
2016	2547	2197	86.3	37,306	33,440	89.6
2017	2520	2191	86.9	38,700	34,430	89.0
2018	2579	2314	89.7	39,651	36,970	93.2
2019	2259	1922	85.1	38,156	35,491	93.0

Source for data: Croatian Health Service Yearbooks 2001–2019 [12,13,14,15,16,17,18,19,20,21,22,23,24,25,26,27,28,29,30].

**Table 2:** Measles–rubella–mumps revaccination in Osijek-Baranja County and Croatia in years 2001 to 2019.

	Osijek-Baranja County 1. Revaccination			Croatia 1. Revaccination		
Year	Scheduled	Vaccinated	%	Scheduled	Vaccinated	%
2001	7971	7902	99.1	101,917	99,395	97.5
2002	7550	7483	99.1	93,404	91,423	97.9
2003	7353	7310	99.4	92,221	90,555	98.2
2004	7429	7369	99.2	86,686	84,837	97.9
2005	3375	3335	98.8	46,153	45,157	97.8
2006	3480	3436	98.7	44,419	43,618	98.2
2007	2993	2942	98.3	42,884	42,086	98.1
2008	3085	3062	99.3	40,733	39,871	97.9
2009	2796	2774	99.2	39,599	38,821	98.0
2010	2785	2759	99.1	39,417	38,547	97.8
2011	2853	2803	98.2	40,410	39,540	97.8
2012	3009	2873	95.5	41,714	40,441	96.9
2013	2841	2787	98.1	41,280	40,098	97.1
2014	2770	2710	97.8	41,454	40,125	96.8
2015	2767	2661	96.2	41,434	39,699	95.8
2016	4111	3965	96.4	43,397	41,647	96.0
2017	2364	2221	94.0	39,630	37,703	95.1
2018	2556	2441	95.5	38,367	36,328	94.7
2019	2046	1921	93.9	36,674	34,745	94.7

Source for data: Croatian Health Service Yearbooks 2001–2019 [12,13,14,15,16,17,18,19,20,21,22,23,24,25,26,27,28,29,30].

**Table 3:** Measles-containing vaccine first dose (%) in Croatia and neighboring countries in the years 2001 to 2019.

Year	BA	HR	MK	ME	RS	SI
2001	92	94	92	*	91	94
2002	89	95	98	*	92	93
2003	84	95	96	*	87	94
2004	88	96	96	*	89	94
2005	90	96	96	*	96	94
2006	90	95	94	90	88	96
2007	96	96	96	90	95	96
2008	84	96	98	89	92	96
2009	93	95	96	86	95	95
2010	92	96	98	90	95	95
2011	89	96	97	91	93	96
2012	94	95	96	90	87	95
2013	*	94	96	88	92	94
2014	89	94	93	76	86	94
2015	83	93	89	64	87	94
2016	68	90	82	47	82	92
2017	69	89	83	58	86	93
2018	68	93	75	42	93	93
2019	*	93	*	*	87	94

* data not available; BA—Bosnia and Herzegovina; HR—Croatia; ME—Montenegro; MK—North Macedonia; RS—Serbia; SI—Slovenia. Source for data: World Health Organization (WHO) statistics from 2001 to 2019 [11].

**Table 4:** Measles-containing revaccination (%) in Croatia and neighboring countries in the years 2001 to 2019.

Year	BA	HR	MK	ME	RS	SI
2001	86	98	94	*	74	98
2002	90	98	95	*	75	*
2003	85	98	97	*	96	*
2004	88	98	95	*	96	*
2005	90	98	95	*	98	*
2006	61	98	96	*	90	99
2007	95	98	95	95	96	98
2008	92	98	95	96	97	99
2009	88	*	97	97	87	98
2010	91	98	99	*	91	96
2011	88	98	98	97	90	96
2012	94	97	96	97	90	96
2013	*	97	96	97	82	95
2014	92	97	96	95	91	94
2015	88	96	93	94	87	96
2016	78	96	93	86	90	93
2017	80	95	97	83	91	94
2018	68	95	94	86	90	94
2019	*	95	*	*	91	94

* data not available; BA—Bosnia and Herzegovina; HR—Croatia; ME—Montenegro; MK—North Macedonia; RS—Serbia; SI—Slovenia; Source for data: World Health Organization (WHO) statistics from 2001 to 2019 [11].

**Table 5:** Measles-containing vaccine 1st dose (%) in European countries from 2001 to 2019.

Year	AT	HU	HR	CZ	DK	DE	IT	PL	FR	BE	UA
2001	79	99	94	*	94	91	77	97	85	82	*
2002	78	99	95	*	99	91	81	98	86	82	*
2003	79	99	95	99	96	92	84	97	87	82	*
2004	74	99	96	97	96	92	86	97	88	82	*
2005	75	99	96	97	95	93	87	98	87	88	*
2006	80	99	95	*	90	94	88	99	89	92	*
2007	79	99	96	98	89	95	90	98	90	92	*
2008	83	99	96	97	*	95	90	98	89	93	*
2009	76	99	95	*	84	96	90	98	70	95	*
2010	*	99	96	*	85	96	91	98	89	95	*
2011	*	99	96	*	87	96	90	98	91	95	*
2012	*	99	95	*	90	97	90	98	90	96	*
2013	*	99	94	*	89	97	90	98	91	96	79
2014	96	99	94	99	90	97	87	97	91	96	56
2015	*	99	93	*	91	97	85	96	90	96	56
2016	95	99	90	98	94	97	87	96	90	96	42
2017	96	99	89	97	97	97	92	94	90	96	86
2018	94	99	93	96	95	97	93	93	*	96	*
2019	*	99	93	92	96	97	94	*	*	96	*

* data not available; AT—Austria; HU—Hungary; HR—Croatia; CZ—Czech Republic; DK—Denmark; DE—Germany; IT—Italy; PL-Poland; FR—France; BE—Belgium; UA—Ukraine; Source for data: World Health Organization (WHO) statistics from 2001 to 2019 [11].

**Table 6:** Measles-containing revaccination (%) in selected European countries from 2001 to 2019.

Year	AT	HU	HR	CZ	DK	DE	IT	PL	FR	BE	UA
2001	34	99	98	97	87	*	*	96	*	*	*
2002	39	99	98	98	92	27	*	97	*	*	*
2003	46	99	98	97	88	53	*	97	*	*	*
2004	47	99	98	97	88	51	*	96	*	*	*
2005	91	99	98	97	91	66	*	90	*	*	*
2006	61	99	98	98	91	77	*	99	*	78	*
2007	56	100	98	98	88	83	*	98	*	78	*
2008	62	100	98	98	*	88	*	97	*	81	*
2009	64	99	*	98	85	89	*	95	*	83	*
2010	*	100	98	98	85	90	*	94	61	83	*
2011	*	100	98	98	86	92	*	95	67	83	*
2012	*	99	97	99	87	92	*	95	72	85	*
2013	*	99	97	99	86	92	84	93	75	85	54
2014	87	100	97	96	84	93	83	95	77	85	57
2015	*	99	96	99	80	93	83	94	79	85	57
2016	89	99	96	93	85	93	82	93	80	85	31
2017	84	99	95	90	88	93	86	93	80	85	84
2018	84	99	95	84	90	93	89	92	83	85	*
2019	*	99	95	*	90	93	88	*	*	85	*

* data not available; AT—Austria; HU—Hungary; HR—Croatia; CZ—Czech Republic; DK—Denmark; DE—Germany; IT—Italy; PL—Poland; FR—France; BE—Belgium; UA—Ukraine; Source for data: World Health Organization (WHO) statistics from 2001 to 2019 [11].

**Table 7:** Number and rate per 1,000,000 measles cases in selected European countries in 2001–2019.

	2001		2002		2003		2004		2005		2006		2007		2008		2009		2010	
	N	R	N	R	N	R	N	R	N	R	N	R	N	R	N	R	N	R	N	R
AL	18	5.9	16	5.2	8	2.6	7	2.3	6	2	68	22.2	22	7.2	0	0	0	0	10	3.3
AT	0	0	0	0	44	5.5	16	2	9	1.1	21	2.6	20	2.4	427	50.9	47	5.6	48	5.7
BE	83	24.8	0	0	44	4.3	61	5.9	25	2.4	15	1.4	58	5.5	98	9.4	33	3.1	40	3.8
BA	0	0	28	6.4	18	4.1	28	6.4	23	5.3	17	3.9	166	37.9	8	1.8	22	5	45	10.3
BG	8	1	0	0	0	0	0	0	3	0.4	1	0.1	1	0.1	1	0.1	2249	298.8	2205	2945.3
HR	8	18	6	1.4	19	4..3	54	12.2	2	0.4	1	0.2	0	0	50	11	2	0.4	7	1.5
CZ	6	0.6	4	0.4	30	29	17	1.7	0	0	6	0.6	2	0.2	2	0.2	5	0.5	0	0
FR	0	0	5185	84.5	0	0	4448	71.5	22	0.4	45	0.7	40	0.7	604	9.8	1544	24.8	5019	80.3
DE	6033	73.6	4665	56.9	778	9.5	122	1.5	778	9.5	2307	27.9	571	6.9	917	11.1	574	6.9	780	9.6
GR	12	1.1	5	0.5	8	0.8	1	0.1	116	10.9	512	46	2	0.2	1	0.1	2	0.2	149	13.3
HU	20	2	0	0	0	0	0	0	2	0.2	1	0.1	0	0	0	0	1	0.1	0	0
IT	799	13.9	18,312	318.7	10,939	190.7	676	11.8	218	3.8	595	10.2	420	7.2	1619	27.5	173	2.9	861	14.6
ME	0	0	0	0	0	0	0	0	0	0	0	0	0	0	0	0	0	0	5	8
PL	133	3.5	34	0.9	48	1.3	11	0.3	14	0.4	120	3.1	43	1.1	100	2.6	109	2.9	10	0.3
PT	21	2.1	7	0.7	7	0.7	1	0.1	7	0.7	0	0	0	0	1	0.1	3	0.3	5	0.5
MK	27	13.3	19	9.4	18	8.9	9	4.4	5	2.5	3	1.5	1	0.5	27	13.3	5	2.5	217	107
RO	10	0.5	14	0.6	9	0.4	117	5.4	5647	254.9	3196	147.8	352	16.3	14	0.7	8	0.4	187	8.8
RS	35	4.7	63	8.4	15	2	11	1.5	2	0.3	2	0.3	201	26.8	2	0.3	1	0.1	20	2.7
SK	0	0	0	0	1	0.2	2	0.4	0	0	0	0	0	0	0	0	0	0	0	0
SI	0	0	0	0	0	0	0	0	0	0	0	0	0	0	0	0	0	0	3	1.5
UA	16,970	350.2	7587	156.6	411	8.5	146	3	2392	49.4	42,724 *	881.7	1005	20.7	48	1	0	0	39	0.8
UK	73	1.2	327	5.5	469	7.8	202	3.4	78	1.3	773	12.9	1004	16.7	1406	23	1166	19	397	6.5
	**2011**		**2012**		**2013**		**2014**		**2015**		**2016**		**2017**		**2018**		**2019**			
	**N**	**R**	**N**	**R**	**N**	**R**	**N**	**R**	**N**	**R**	**N**	**R**	**N**	**R**	**N**	**R**	**N**	**R**		
AL	28	9.9	9	3.2	0	0	0	0	0	0	17	6	12	4.2	1469	518.8	488	172.3		
AT	99	12	19	2.3	75	8.9	112	13.3	309	36	27	3.1	95	10.8	77	8.7	151	17		
BE	555	51	43	3.9	38	3.4	70	6.3	46	4.1	78	6.9	367	32.3	117	10.3	496	43.3		
BA	10	2.3	22	5	0	0	3000	849.6	1677	474.9	133	37.7	18	5.1	62	17.6	1404	397.6		
BG	157	21	1	0.1	16	2.2	0	0	0	0	1	0.1	165	23.2	13	1.8	1235	176.4		
HR	12	2.8	2	0.5	1	0.2	14	3.3	219	51.8	4	1	7	1.7	23	5.6	52	12.8		
CZ	17	1.6	22	2.1	14	1.3	222	21.1	9	0.9	7	0.7	146	13.8	207	19.5	590	55.4		
FR	15,206	234	859	13.2	272	4.2	267	4.1	364	5.5	79	1.2	518	7.8	2919	43.6	2636	39.3		
DE	1609	20	167	2	1772	21.7	446	5.4	2646	30.4	326	4	929	11.3	543	6.6	514	6.2		
GR	40	4	3	0.3	3	0.3	1	0.1	1	0.1	0	0	967	89.8	2293	213.5	45	4.2		
HU	5	0.5	2	0.2	1	0.1	0	0	0	0	0	0	36	3.7	14	1.4	23	2.4		
IT	5181	85	682	11.2	2216	36.4	1676	28.1	256	4.2	861	14.2	5399	89.1	2686	44.4	1620	26.8		
ME	5	8	0	0	0	0	0	0	0	0	0	0	0	0	200	319.9	0	0		
PL	38	1	61	1.6	86	2.2	110	2.9	48	1.3	133	3.5	63	1.7	340	9	1423	37.5		
PT	2	0.2	7	0.7	1	0.1	0	0	0	0	0	0	34	3.3	171	16.6	10	1		
MK	701	346.6	7	3.5	4	2	116	57.4	1	0.5	0	0	19	9.4	64	31.6	1337	661		
RO	4015	187	3843	179.5	1074	50.3	53	2.6	7	0.4	2435	123.1	9076	462	6398	327.6	1706	87.9		
RS	370	51.5	0	0	1	0.1	37	5.1	383	53.3	11	1.5	721	100.3	5076	706.3	22	3.1		
SK	2	0.4	1	0.2	0	0	0	0	0	0	0	0	6	1.1	565	103.8	319	58.5		
SI	22	11	2	1	1	0.5	52	25.3	18	8.7	1	0.5	8	3.9	9	4.4	48	23.1		
UA	1333	29.2	12,746	279.5	0	0	0	0	105	2.3	102	2.2	4782	1049	53,219 *	1167.1	57,282 *	1256.2		
UK	1083	17	1902	30.4	1900	30.7	133	2.1	92	1.4	571	8.7	280	4.3	953	14.4	882	13.2		

N—number of measles cases; AL—Albania; AT—Austria; BE—Belgium; BA—Bosnia and Herzegovina; BG—Bulgaria; HR—Croatia; CZ—Czech Republic; FR—France; DE—Germany; GR—Greece; HU—Hungary; IT—Italy; ME—Montenegro; PL—Poland; PT—Portugal; MK—North Macedonia; RO—Romania; RS—Serbia; SK—Slovakia; SI—Slovenia; UA—Ukraine; UK—United Kingdom of Great Britain and Northern Ireland; * *p* < 0.005 2006, 2018 and 2019 Ukraine vs. all other countries; Source for data: World Health Organization Regional Office for Europe [31].

## Data Availability

Not applicable.
